# Expression of intestinal transporter genes in beagle dogs

**DOI:** 10.3892/etm.2012.777

**Published:** 2012-10-30

**Authors:** SOO-MIN CHO, SUNG-WON PARK, NA-HYUN KIM, JIN-A PARK, HEE YI, HEE-JUNG CHO, KI-HWAN PARK, INGYUN HWANG, HO-CHUL SHIN

**Affiliations:** 1 Department of Veterinary Pharmacology and Toxicology, College of Veterinary Medicine, Konkuk University, Seoul 143-701;; 2 Toxicology and Chemistry Division, Animal Plant and Fisheries Quarantine and Inspection Agency, Anyang 430-824;; 3 Department of Food Science & Technology, Chung-Ang University, Ansung 456-756;; 4 Food Chemical Residues Division, Korea Food & Drug Administration, Osong 363-951, Republic of Korea

**Keywords:** gene expression, transporters, intestine, dog

## Abstract

This study was performed to produce a transcriptional database of the intestinal transporters of beagle dogs. Total RNA was isolated from the duodenum and the expression of various mRNAs was measured using GeneChip^®^ oligonucleotide arrays. A total of 124 transporter genes were detected. Genes for fatty acid, peptide, amino acid and glucose and multidrug resistance/multidrug resistance-associated protein (MDR/MRP) transport were expressed at relatively higher levels than the other transporter types. The dogs exhibited abundant mRNA expression of the fatty acid transporters (fatty acid binding proteins, FABPs) FABP1 and FABP2, the ATP-binding cassettes (ABCs) ABCB1A and ABCC2, the amino acid/peptide transporters SLC3A1 and SLC15A1, the glucose transporters SLC5A1, SLC2A2 and SLC2A5, the organic anion transporter SLC22A9 and the phosphate transporters SLC20A1 and SLC37A4. In mice, a similar profile was observed with high expression of the glucose transporters SLC5A1 and SLC2As, the fatty acid transporters FABP1 and FABP2, the MDR/MRP transporters ABCB1A and ABCC2 and the phosphate transporter SLC37A4. However, the overall data reveal diverse transcriptomic profiles of the intestinal transporters of dogs and mice. Therefore, the current database may be useful for comparing the intestinal transport systems of dogs with those of mice to better evaluate xenobiotics.

## Introduction

Intestinal drug transporters have great potential for drug absorption (1,2). They may serve as either drug targets or drug delivery systems. It is generally assumed that at least 5% of all human genes are associated with transporters which is consistent with the biological significance of transporters and their roles in cell homeostasis. The identification and characterization of drug transporters has provided a scientific basis for understanding drug delivery and disposition, the molecular mechanisms of drug interactions and inter-individual/inter-species differences (3). Various types of xenobiotic or drug transporters have been identified as being important as barriers against toxic compounds and influx pumps to take up nutrients into the body. Since these xenobiotic transporters generally have a wide range of recognition specificities and accept various types of compounds as substrates, the localization and functional expression of such transporters may be a critical factor in the disposition and subsequent biological activity of therapeutic agents.

Dogs have emerged as a primary species for the study of biology and human diseases (4). The organization of the dog genome has been studied extensively in the last ten years. With advances in genomics, microarray technology has been used to identify tissue-specific genes, including intestinal transporters. It is now accepted that the process of drug absorption in the intestine is highly associated with the functional expression of intestinal transporters (5). The current study was therefore carried out to generate a gene expression database of transporters in the canine duodenum.

## Materials and methods

### Materials

The TRIzol^®^ reagent and SuperScript Choice System cDNA synthesis kit were purchased from Invitrogen (Carlsbad, CA, USA). The BioArray high-yield RNA transcript labeling kit was obtained from Enzo Biochem (New York, NY, USA). The RNeasy kit was supplied by Qiagen (Valencia, CA, USA). The Canine 2.0 and Mouse 430A 2.0 GeneChips were provided by Affymetrix (Santa Clara, CA, USA). GeneChip hybridization and scanning were performed at the Seoulin Molecular Biology Technique Center (Seoul, Korea).

### Animals

Beagle dogs (4 males, 5.5–7.6 kg; Marshall BioResource, Beijing, China) were housed in a controlled semi-barrier system room at Chemon Co. (Yongin, Korea). The animals were considered to be healthy based on clinical examination (Korea Food and Drug Administration Guide for the Care and Use of Laboratory Animals, 2009). ICR mice (30–40 g) were obtained from Orient Bio Co. (Seoul, Korea) and housed in a controlled animal room at Konkuk University (Seoul, Korea). The animals were fed solid pellets and provided with water *ad libitum*. All procedures were approved by the Konkuk University Institutional Animal Care and Use Committee.

### RNA isolation

Mucosal tissues obtained from dog and mouse duodenums were immediately scraped with a clean glass slide, transferred to a new frozen vial and dipped into liquid N_2_. Tissue (∼100 mg) was added to 1 ml TRIzol reagent and homogenized with a razor on ice. The homogenate was transferred to a new tube and then 200 μl chloroform was added to the TRIzol mixture. Following centrifugation at 12,500 rpm for 15 min at 4°C, the aqueous phase was transferred to a new tube. The RNA was then precipitated with 500 μl isopropanol and washed with 80% ethanol. The RNA was further purified with an RNeasy Mini kit (Qiagen) according to the manufacturer’s instructions. The concentration of the purified RNA was measured at 260 nm and 5 μg purified RNA was mixed with RNA loading buffer and heated at 75°C for 15 min. After cooling on ice for 5 min, the RNA was loaded onto 1% agarose/formaldehyde gel in 1X MOPS buffer. The gel was run at 80–100 V for 50 min and the presence of two sharp 18S and 28S bands was confirmed under UV light.

### Microarray assay

Once the total RNA samples were prepared, probe synthesis, hybridization, detection and scanning were performed according to the standard instructions of the manufacturer (Affymetrix, Inc.). The cDNA was synthesized using the One-Cycle cDNA Synthesis kit. Single-stranded cDNA was synthesized using Superscript II reverse transcriptase and T7-oligo(dT) primers at 42°C for 1 h. Double-stranded (ds) cDNA was obtained through a reaction using DNA ligase, DNA polymerase I and RNase H at 16°C for 2 h, followed by T4 DNA polymerase at 16°C for 5 min. After clean up with a Sample Cleanup Module (Affymetrix, Inc.), ds-cDNA was used for *in vitro* transcription (IVT). cDNA was transcribed using the GeneChip IVT Labeling kit (Affymetrix, Inc.) in the presence of biotin-labeled CTP and UTP. The resulting biotin-labeled IVT-RNA was again purified with a Sample Cleanup Module (Affymetrix, Inc.) and subsequently fragmented. Fragmented cRNA was hybridized at 45°C for 16 h according to the manufacturer’s instructions. Following hybridization, the arrays were washed in a GeneChip Fluidics Station 450 with a non-stringent wash buffer at 25°C and then by a stringent wash buffer at 50°C. The arrays were then stained with a streptavidin-phycoerythrin complex. After staining, the intensities were determined with a GeneChip scanner. The duodenal mRNA expression profile obtained from microarray data analyses for SLC15A1 was validated using semiquantitative RT-PCR. The RT-PCR assay was performed as described previously (6). The pattern of SLC15A1 mRNA expression in the individual biopsies determined by RT-PCR was similar to that observed in the microarray data.

### Data analysis

Official symbols and gene names were used in accordance with the symbol and name lists approved by the Human Genome Organization (HUGO) Gene Nomenclature Committee (http://www.genenames.org). Data analysis was performed using GeneSpring 7.2 software (Silicon Genetics, Redwood City, CA, USA). The numeric data were extracted from DAT images and normalized using Microarray Suite software. Gene function analysis was performed using the gene ontology-mining tool of NetAffx, which is based on the Gene Ontology database (http://www.geneontology.org). GeneSpring also uses data from public genomics databases to build gene ontologies based on annotation information. For the present GeneChip probe array study, the data for each gene represented data from 11–20 probe pairs, each ∼25 bp in length. The overall target-specific intensity was measured as the difference between the intensity of the perfectly matched and mismatched probes. For normalization, data from each expression array were scaled so that the overall fluorescence intensity across each chip was equivalent (average target intensity set at 500). The One-Sided Wilcoxon Signed Rank test was employed to generate the detection P-value. If the overall intensity of the perfect match was significantly larger than that of the mismatch, the detection P-value was small. The probed gene set was considered to be present if the P-value was <0.04. If the P-value was >0.06, the probe set was considered to be absent. The change algorithm generated a change P-value and an associated fold-change value. The second algorithm gave a quantitative estimate of the change in gene expression in the form of a signal log ratio. The level of gene expression was considered to be increased if its change P-value was <0.0025 and the gene expression was considered to be decreased if its change P-value was >0.9975.

## Results

### Sequence analysis

A total of 43,035 sequences from the dog duodenum were analyzed and 60% exhibited >1-fold changes. As shown in Table I, the total numbers of detected transporter genes were 124 in the dog duodenum and 130 in the mouse intestine. Among the transporter groups, the expression levels of fatty acid, peptide, amino acid, glucose and multidrug resistance/multidrug resistance-associated protein (MDR/MRP) transporter genes were relatively higher than those of other transporter gene groups in the two species.

### Glucose transporters

**Figure 1:**
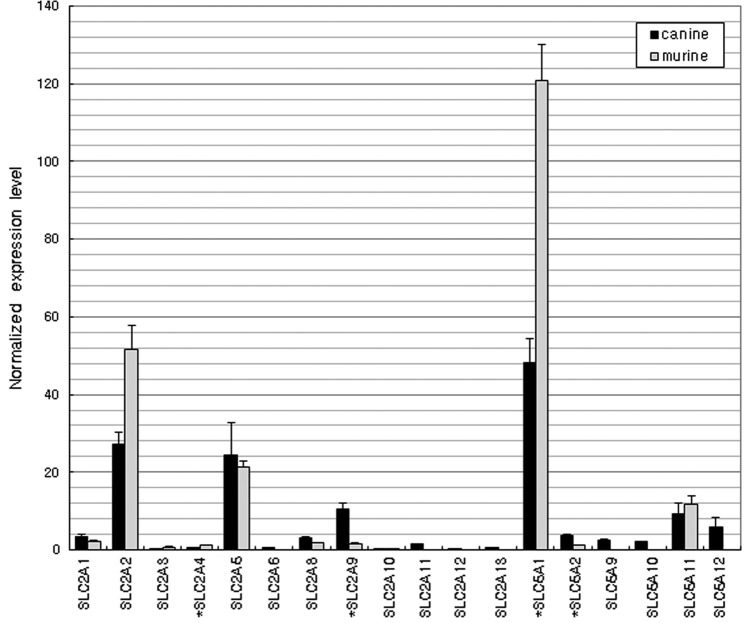
Comparative gene expression of various glucose transporters in the intestines of dogs and mice (n=4). Expression levels were measured as the intensity of the hybridization signal in a GeneChip array and normalized using GeneSpring 7.2. ^*^>2-fold difference in gene expression between the two species (t-test, P<0.01). SLC2, solute carrier family 2 (GLUTs, facilitated glucose transporters); SLC5, solute carrier family 5 (SGLTs, sodium/glucose cotransporters).

Fig. 1 shows the similar expression profiles of the glucose transporter genes in the dogs and mice. The majority of the SLC2 family (facilitated glucose transporters, GLUTs) and SLC5 family (sodium/glucose cotransporters, SGLTs) genes were expressed. SLC2A2, SLC2A5 and SLC5A1 were the dominantly expressed genes in the two species.

### Amino acid and peptide transporters

**Figure 2:**
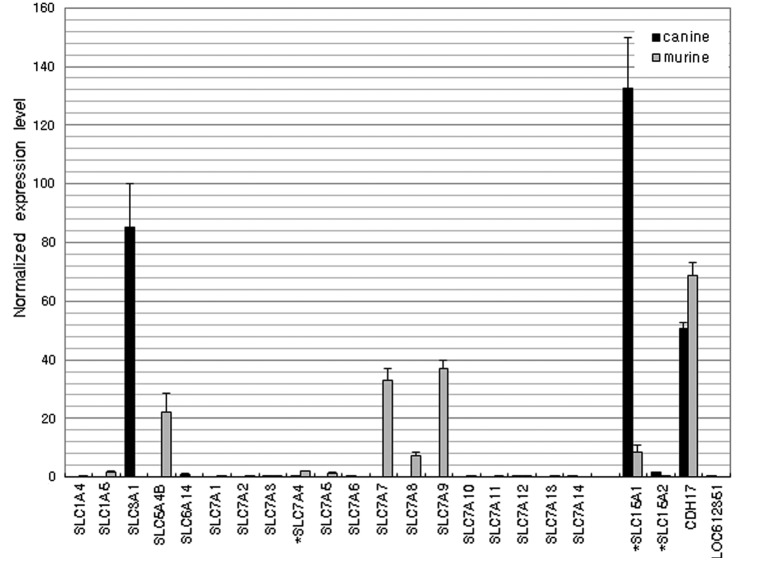
Comparative expression of various amino acid and peptide transporter genes in the intestines of dogs and mice (n=4). SLC1, solute carrier family 1 (neuronal/epithelial high affinity glutamate transporters, system Xag); SLC5, solute carrier family 5 (sodium/glucose cotransporters); SLC7, solute carrier family 7 (cationic amino acid transporters, y+ system); CDH17, cadherin 17/Hpt1, SLC15 solute carrier family 15 (oligopeptide transporters); LOC612351, similar to peptide/histidine transporter PHT2.

Fig. 2 shows the expression levels of various amino acid and peptide transporter genes in the duodenums of the dogs and mice. SLC3A1 and SLC15A1 were the dominantly expressed transporters in dogs, while SLC7A9 and SLC7A7 were highly expressed in mice. The expression of SLC15A1 was >15-fold higher in dogs than in mice (P<0.01).

### Fatty acid transporters

**Figure 3:**
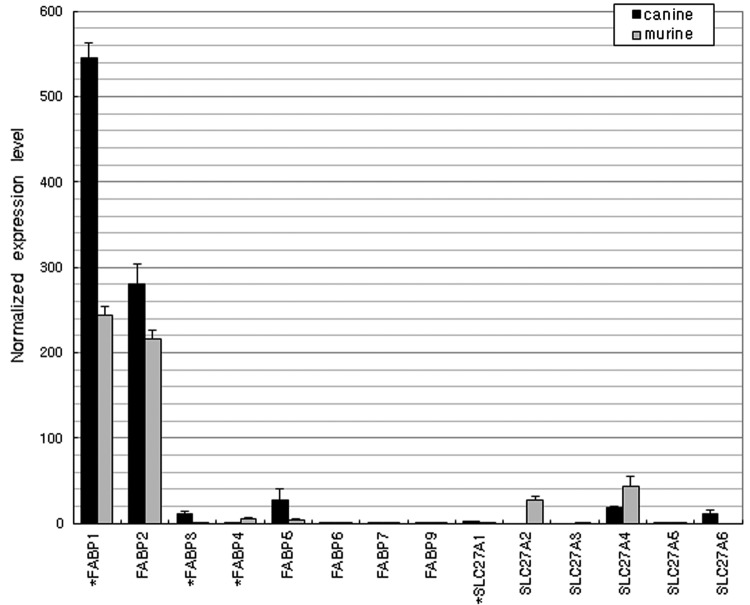
Comparative expression of various fatty acid transporter genes in the intestines of dogs and mice (n=4). FABP, fatty acid binding protein; SLC27, solute carrier family 27 (fatty acid transporters).

The expression levels of fatty acid transporter genes are shown in Fig. 3. The overall expression levels of fatty acid transporter genes were relatively high compared with those of the other transporter groups (Table I). Among the fatty acid binding proteins (FABPs), FABP1 and FABP2 were the most dominantly expressed in both species.

### Nucleobase and nucleoside transporters

**Figure 4:**
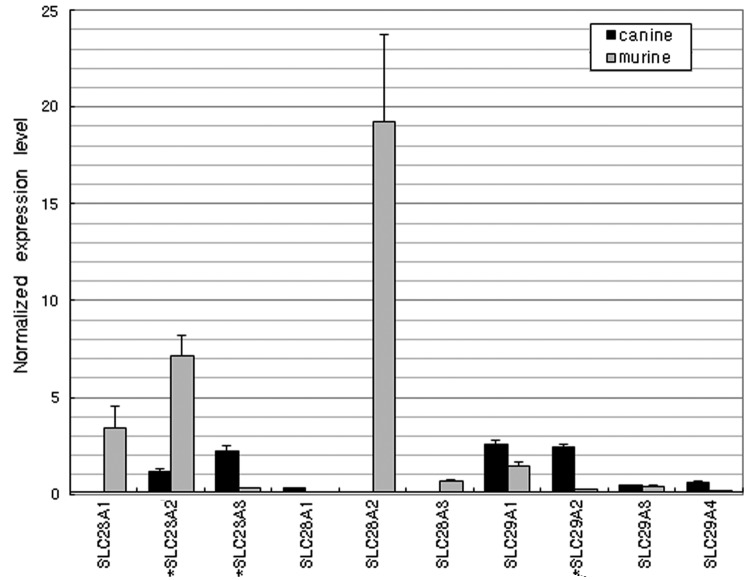
Comparative expression of various nucleobase and nucleoside transporter genes in the intestines of dogs and mice (n=4). SLC23, solute carrier family 23 (nucleobase transporters); SLC28, solute carrier family 28 (sodium-coupled nucleoside transporters, CNTs); SLC29, solute carrier family 29 (nucleoside transporters).

As nucleobase transporters, SLC23A1 and SLC23A2 were highly expressed in mice but poorly expressed in dogs (Fig. 4). Among the nucleoside transporter genes, SLC28A2 (a sodium-coupled nucleoside transporter) was highly expressed in mice but not in dogs. Another family of nucleoside transporters, including SLC29A1 and SLC29A2, was more highly expressed in dogs than in mice.

### MDR and MRPs

**Figure 5:**
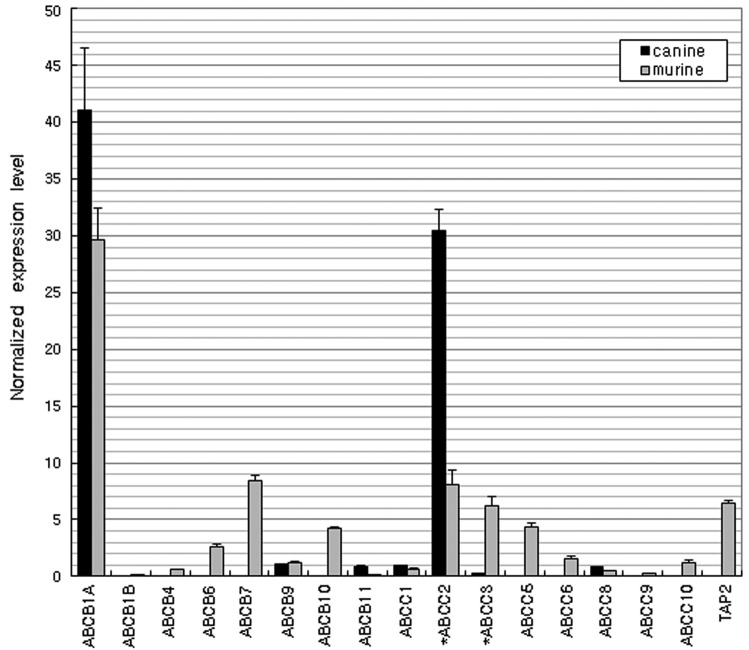
Comparative expression of various MDR and MRP genes in the intestines of dogs and mice (n=4). MDR/MRP, multidrug resistance/multidrug resistance-associated protein; ABCB, ATP-binding cassette sub-family B (MDR/TAP); ABCC, ATP-binding cassette sub-family C (CFTR/MRP); TAP2, transporter 2 ATP-binding cassette sub-family B (MDR/TAP).

Fig. 5 shows the expression levels of ATP-binding cassette (ABC) genes. Similarly high expression of ABCB1A (MDR/TAP) and ABCC2 (CFTR/MRP) were observed in both species.

### Organic anion and cation transporters

**Figure 6:**
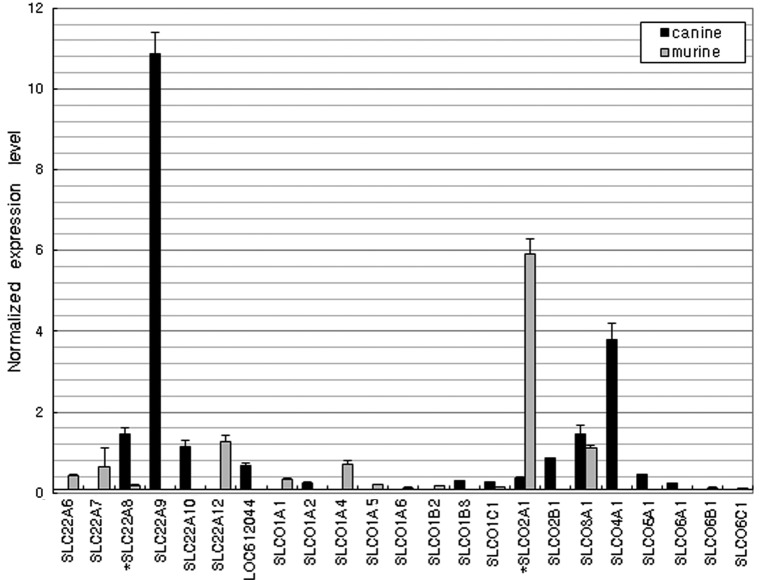
Comparative expression of various organic anion transporter genes in the intestines of dogs and mice (n=4). SLC22, solute carrier family 22 (organic cation transporters); LOC612044, similar to SLC22A20; SLCO, solute carrier organic anion transporter family.

**Figure 7:**
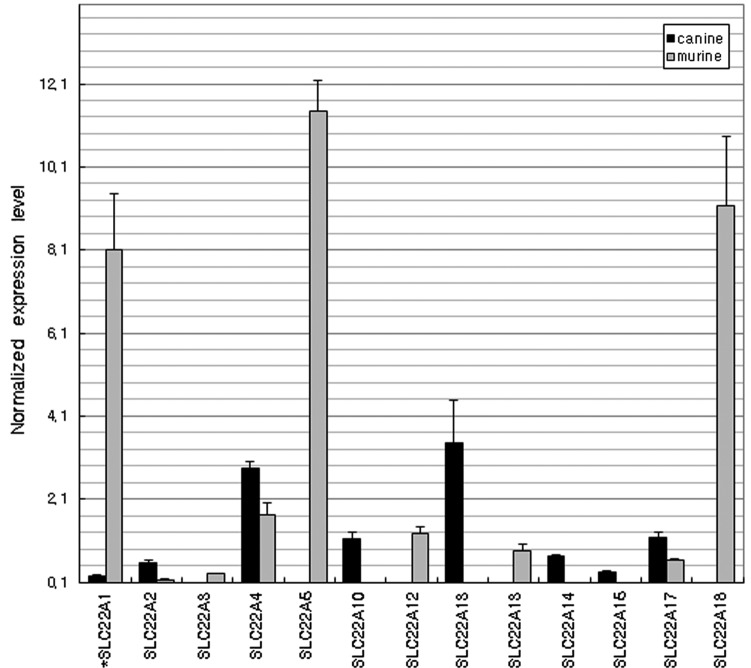
Comparative expression of various organic cation transporter genes in the intestines of dogs and mice (n=4). SLC22, solute carrier family 22 (organic cation transporters).

Fig. 6 shows an interspecies variation in the expression levels of organic anion transporters. The most highly expressed anion transporter genes in the dog duodenum were SLC22A9 and SLCO4A1 and the most strongly expressed gene in mice was SLCO2A1. Of the organic cation transporters (Fig. 7), SLC22A1, SLC22A5 and SLC22A18 were dominantly expressed in the mice although their expression levels were negligible in the dogs. However, the expression of SLC22A13 in dogs was >30-fold higher than that in mice (P<0.01).

### Phosphate transporters

**Figure 8:**
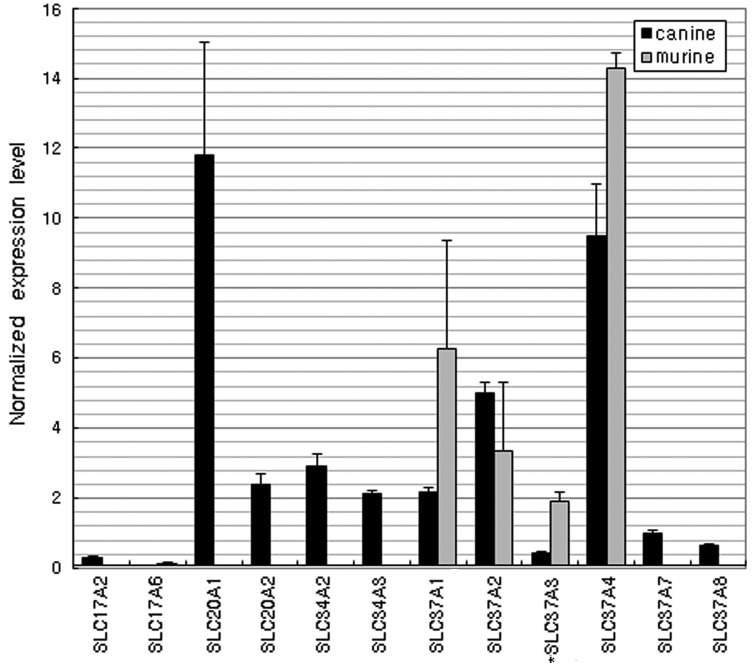
Comparative expression of various phosphate transporter genes in the intestines of dogs and mice (n=4). SLC17, solute carrier family 17 (sodium phosphate transporters); SLC20, solute carrier family 20 (phosphate transporters); SLC34, solute carrier family 34 (sodium phosphate transporters); SLC37, solute carrier family 37 (glycerol-3-phosphate transporters).

Among the various phosphate transporters, the SLC17 (sodium phosphate transporter), SLC20 (phosphate transporter), SLC34 (sodium phosphate transporter) and SLC37 (glycerol-3-phosphate transporter) family genes were detected in the dogs (Fig. 8). Only 4 SLC37 family genes, SLC37A1, SLC37A2, SLC37A3 and SLC37A4, were expressed in the mice. The most highly expressed genes were SLC20A1 in dogs and SLC37A4 in mice (P<0.01).

## Discussion

The completion of the DNA sequencing of the human, mouse, dog and rat genomes and knowledge of cross-species gene homologies enables the study of differential gene expression in animal models (7). The characterization of tissue-specific genes, such as intestinal transporters, has the potential to greatly enhance the understanding of the bioavailability of oral drugs (8–13). It is now accepted that the process of drug absorption in the intestine is highly associated with the functional gene expression of intestinal transporters (8). However, little is known about the transporter genes that are transcribed in the dog intestine.

Membrane transport is a critical process for the intestinal absorption of glucose, one of the primary energy sources for various physiological and pharmacological functions (14). All glucose transporters have significant homologies but they function differently to transport various sugars or sugar-associated compounds from the intestine to the systemic circulation with interspecies diversities (15). The data of the present study revealed that the three glucose transporters SLC2A2 (GLUT2), SLC2A5 (GLUT5) and SLC5A1 (SGLT1) are similarly dominantly expressed in dogs and mice. In humans, SLC5A1 transports glucose from the intestinal lumen to the cytosol and SLC2A2 transports glucose from the cytosol to the blood. Intestinal glucose absorption by the apical SLC2A2 pathway may be 3- to 5-times greater than that by SGLT1 at high concentrations of sugar (16). SLC2A5 preferentially transports fructose rather than glucose (17).

The classification of amino acid transporters is well defined according to their substrate specificity and tissue distribution (18). The amino acid transporters have highly restrictive substrate specificities (19). The intestinal uptake of certain anticancer drugs, such as the toxicants gabapentin, pregabalin, melphalan, baclofen, D-cycloserine and methyl-mercury L-cysteine complex, and other drugs is known to be mediated specifically by these transporters. In the present study, a significant difference was observed between the expression of the transporters in the two species. SLC3A1 was dominantly expressed in dogs and SLC7A9 and SLC7A7 (cationic amino acid transporters) were detected at high levels in mice. Previous studies have demonstrated that amino acid transporters are good targets for improving the oral bioavailability of amino acid-associated drugs. The peptide transporter SLC15A1 (PEPT1) was expressed in both species but its expression level was 15-fold higher in dogs than in mice. The proton-coupled peptide transporter is responsible for the absorption of small peptides arising from the digestion of dietary proteins (20). It is well known that SLC15A1 is responsible for the uptake of a number of peptide-like drugs, including β-lactam antibiotics, angiotensin converting enzyme inhibitors, renin inhibitors, antitumor or antiviral agents, thrombin inhibitors, a dopamine receptor antagonist and amino acid prodrugs (21). Kim *et al*(22) also reported the notable expression of SLC15A1 in the rat intestine.

Fatty acids are absorbed by passive diffusion. However, numerous studies have demonstrated that various transporters are involved in fatty acid absorption (23). Intestinal enterocytes contain high concentrations of two cytosolic FABPs, FABP1 and FABP2, which are hypothesized to be involved in cellular fatty acid trafficking. It was suggested that FABP2, but not FABP1, may directly extract fatty acids from membranes (24). The present results reveal that the two FABP genes are the principal fatty acid transporters expressed in the intestines of dogs and mice with similar expression trends in both species. Schaffer and Lodish (25) identified a membrane protein which they termed fatty acid transport protein (FATP) from mouse adipocytes. However, in the present study, the expression of FATP was not particularly strong in either species.

Aside from the crucial roles of purines and pyrimidines in DNA and RNA synthesis, they are also significant components of a number of important biomolecules (26). Therefore, there is a specific transport system associated with active transporters for delivering nucleobases or nucleosides into cells. In the present study, a considerable difference in the expression of nucleobase and nucleoside transporters was observed between dogs and mice. In mice, the nucleobase transporters SLC23A1 and SLC23A2 and nucleoside transporter SLC28A2 (sodium-coupled nucleoside transporters) were highly expressed but these genes were poorly expressed in dogs. By contrast, the nucleoside transporters SLC29A1 and SLC29A2 were highly expressed in dogs. Nucleobase and nucleoside analogs are widely used in the treatment of neoplasms and viral infections (27,28).

Typical efflux transporters involve MRPs and MDRs, which are members of the ABC superfamily. These proteins translocate a wide variety of substrates, including sugars, amino acids, nucleosides, lipids, bile salts, metal ions, peptides, proteins and a large number of hydrophobic compounds and metabolites across extra- and intracellular membranes (29,30). In the present study, ABCB1A and ABCC2 (MRP2) were the most highly expressed genes in the two species. Previous studies revealed that ABCC2 accepts glutathione conjugates, sulfate conjugates, glucuronides and non-conjugated organic anions, pravastatin, vinblastine, temocaprilat, BQ-123, methotrexate, irinotecan and HIV protease inhibitors, including saquinavir, ritonavir and indinavir. ABCC3 (MRP3) mediates the transport of glucuronide conjugates, taurocholate, glycocholate and methotrexate. Differential expression of ABCC3 was observed between the two species but the expression levels of ABCC3 were lower than those of ABCC2.

Organic anion transporters serve as the efflux system for a number of endogenous compounds, anionic drugs, environmental substances and their metabolic products, which are usually harmful to the body. In particular, these families are known to affect the pharmacokinetics and drug-drug interactions of various drugs (31). In the present study, SLC22A9 and SLCO4A1 in dogs and SLCO2A1 in mice were dominantly expressed and the overall levels of expression were higher in dogs than in mice. SLCO4A1 mediates the sodium-independent transport of organic anions, including the thyroid hormones triiodo-L-thyronine and thyroxine. Organic cation transporters in the liver, kidney and intestine are critical for the absorption and elimination of a number of endogenous amines as well as a wide range of drugs and environmental toxins. In the present study, the SLC22 family gene expression profile was markedly different between the dogs and mice. The expression of SLC22A13 was more than 30-fold higher in dogs than in mice. SLC22A13 was originally known as an organic cation transporter but Bahn *et al*(32) renamed it OAT10 (organic anion transporter) since it is able to transport various anionic compounds, including nicotinate, p-aminohippurate and urate.

In the analysis of phosphate transporters, the data reveal that members of the SLC17, SLC20, SLC34 and SLC37 families were expressed in the dogs, while only SLC37 family genes, including SLC37A1, SLC37A2, SLC37A3 and SLC37A4, were expressed in the mice. The most highly expressed genes in the dogs and mice were SLC20A1 and SLC37A4, respectively. SLC20A1 is a ubiquitously expressed sodium-phosphate symporter that plays a fundamental housekeeping role in the maintenance of phosphate homeostasis. SLC20A1 may also function as a retroviral receptor as it confers susceptibility to certain viral infections in human cells.

Although similar profiles were observed for several transporters in both species, including the glucose transporters SLC5A1 and SLC2As, the fatty acid transporters FABP1 and FABP2, the efflux transporters ABCB1A and ABCC2 and the phosphate transporter SLC37A4, overall, the data of the present study reveal markedly different transcriptomic profiles for the intestinal transporters of dogs and mice. The dog is an extremely important animal species, not only as a laboratory animal, but also as a major animal in veterinary medicine. However, genetic information has not been explored extensively for the dog in comparison with other model animals. Therefore, the database generated in the present study may be useful for comparing the intestinal xenobiotic transport systems of dogs and other animals.

## Figures and Tables

**Table I:** Numbers of transporter genes expressed in dog and mouse intestines.

	Dog		Mouse	
Transporter cluster	Number	Expression level (mean ± SD)	Number	Expression level (mean ± SD)
Glucose transporters	18	8.1±12.8	11	19.5±37.0
Amino acid transporters	7	12.6±31.9	15	7.1±12.7
Peptide transporters	4	46.3±62.1	3	25.8±37.5
Fatty acid transporter	12	75.0±167.8	13	41.9±85.0
Mitochondrial solute carrier	10	16.6±50.5	16	23.8±42.3
Nucleoside transporters	5	1.7±0. 8	6	3.6±3.4
Nucleobase transporter	2	1.3±1.1	3	3.7±7.6
MDRs/MRPs	7	10.8±17.3	17	4.5±7.1
Organic anion transporters	13	1.7±2.9	14	0.8±1.5
Organic cation transporters	8	1.3±1.2	9	3.7±4.5
Phosphate transporters	11	3.5±3.8	5	5.2±5.56
Sodium/hydrogen exchangers	8	1.0±0.8	2	1.8±0. 5
Sulfate transporter	2	6.1±7.3	2	1.6±1.1
Zinc Transporters	17	2.9±4.2	14	6.7±5.2
Total	124	–	130	–

MDRs/MRPs, multidrug resistance/multidrug resistance-associated proteins.
